# Complete Genome Assembly of Multidrug-Resistant Yersinia enterocolitica Y72, Isolated in Sweden

**DOI:** 10.1128/MRA.00264-21

**Published:** 2021-05-13

**Authors:** Philip A. Karlsson, Tifaine Hechard, Cecilia Jernberg, Helen Wang

**Affiliations:** a Department of Medical Biochemistry and Microbiology, Uppsala University, Uppsala, Sweden; b Public Health Agency of Sweden, Solna, Sweden; University of Maryland School of Medicine

## Abstract

Here, we report the complete genome sequence of a Swedish clinical strain of Yersinia enterocolitica, Y72. With emerging *Yersinia* outbreaks circulating in Nordic countries, the Y72 genome will provide more insights on the genetic relatedness and antibiotic resistance dissemination in future studies.

## ANNOUNCEMENT

Yersinia enterocolitica is a foodborne pathogen and one of the most commonly reported zoonotic infections in Europe (1–3). In the spring of 2019, the Swedish Public Health Agency (PHAS) and Statens Serum Institut in Denmark independently recognized an outbreak with Y. enterocolitica 4/O:3 (4). The PHAS requested isolates from the clinical microbiological laboratories for epidemiological typing to investigate the national outbreak. Y72 was one of these isolates. Screening for *Yersinia* isolates encompasses cultivation of a feces sample on cefsulodin-irgasan-novobiocin (CIN) agar at 30°C for 1 to 2 days. Isolates are confirmed using matrix-assisted laser desorption ionization–time of flight (MALDI-TOF) mass spectrometry. Y72 was grown on CIN agar before being inoculated in 1 ml Tryptic soy broth at 26°C with shaking at 200 rpm overnight. Genomic DNA was extracted with the MasterPure complete DNA and RNA purification kit (MC85200; Epicentre). Illumina and Nanopore sequencing libraries were prepared using the NEBNext Ultra DNA library prep kit (E7370L; New England BioLabs) and rapid barcoding sequencing (SQK-RBK004; Oxford Nanopore Technologies), respectively. DNA libraries were sequenced using the Illumina HiSeq 2500 platform by Novogene and a MinION instrument by Oxford Nanopore Technologies (5–7). Default parameters were used for all software in the manuscript unless otherwise specified. MinION sequencing was run with an R9 12-type flow cell (FLO-MIN106D) in the MinKNOW software with base calling. A total of 2 × 5,364,739 paired-end reads (150 bp in length) were sequenced with Illumina, and 8,046 reads (average length, 11,298 bp) were sequenced with Nanopore. Illumina reads were quality controlled by Novogene (effective rate, 99.88%; error rate, 0.03%; Q20%, 98.02%), and Nanopore reads were corrected downstream using the Illumina reads in CLC Genomics Workbench 21.0.4 (Qiagen).

Raw sequences were trimmed and filtered (quality score limit, 5%) in CLC, and Nanopore reads were *de novo* assembled (De Novo Assemble Long Reads tool; CLC), resulting in three individual contigs, including 1 circular chromosome (4,567,080 bp), the *Yersinia* pYV plasmid (71,760 bp), and a novel plasmid, pYE-tet (5,677 bp). Trimmed Illumina raw reads were mapped against the three Nanopore contigs (CLC), giving an average chromosomal coverage of 322× (GC content, 47%), a pYV coverage of 632× (GC content, 44%), and a pYE-tet coverage of 5,155× (GC content, 38%). The genome was deemed complete following the comparison and localization of replication origins from alignment (Whole Genome Alignment plugin; CLC) to a Y. enterocolitica strain Y11 chromosome (GenBank accession no. FR729477) and pYV plasmid (FR745874). The replication origin of pYE-tet was identified following alignment to Actinobacillus pleuropneumoniae strain MV780, plasmid p780 (MH457196).

The genome was annotated using the NCBI Prokaryotic Genome Annotation Pipeline (PGAP), which identified 4,254 genes and 7 rRNA, 66 tRNA, and 10 noncoding RNA (ncRNA) genes, also confirming the suggested biovar and serotype (8). PGAP (version 5.0) applied the best-placed reference protein set (GeneMarkS-2+). The full genome was analyzed for antimicrobial resistance genes using ResFinder 4.1 (https://cge.cbs.dtu.dk/services/ResFinder/) (9, 10). ResFinder identified multidrug resistance genes *vatF*, *aadA1*, *catA1*, *blaA*, and *sul1* on the chromosome, as well as *tetB* on the pYE-tet plasmid.

### Data availability.

The sequence data of the fully assembled Y. enterocolitica Y72 isolate are available at NCBI under BioProject PRJNA698800, BioSample SAMN17740595, and accession JAFBLR000000000.1. The raw reads are also available at SRR13960196 (Illumina) and SRR13960195 (Nanopore).

## References

[1] Scallan E, Hoekstra RM, Angulo FJ, Tauxe RV, Widdowson M-A, Roy SL, Jones JL, Griffin PM. 2011. Foodborne illness acquired in the United States—major pathogens. Emerg Infect Dis 17:7–15. doi:10.3201/eid1701.P11101. 21192848 PMC3375761

[2] Batz MB, Hoffmann S, Morris JG, Jr. 2012. Ranking the disease burden of 14 pathogens in food sources in the United States using attribution data from outbreak investigations and expert elicitation. J Food Prot 75:1278–1291. doi:10.4315/0362-028X.JFP-11-418. 22980012

[3] EFSA and ECDC. 2019. The European Union One Health 2018 zoonoses report. EFSA J 17:e05926. doi:10.2903/j.efsa.2019.5926. 32626211 PMC7055727

[4] Espenhain L, Riess M, Muller L, Colombe S, Ethelberg S, Litrup E, Jernberg C, Kuhlmann-Berenzon S, Lindblad M, Hove NK, Torpdahl M, Mork MJ. 2019. Cross-border outbreak of Yersinia enterocolitica O3 associated with imported fresh spinach, Sweden and Denmark, March 2019. Euro Surveill 24:1900368. doi:10.2807/1560-7917.ES.2019.24.24.1900368. PMC6582516 31213223

[5] Jain M, Olsen HE, Paten B, Akeson M. 2016. The Oxford Nanopore MinION: delivery of nanopore sequencing to the genomics community. Genome Biol 17:239. doi:10.1186/s13059-016-1103-0. 27887629 PMC5124260

[6] Lemon JK, Khil PP, Frank KM, Dekker JP. 2017. Rapid nanopore sequencing of plasmids and resistance gene detection in clinical isolates. J Clin Microbiol 55:3530–3543. doi:10.1128/JCM.01069-17. 29021151 PMC5703817

[7] George S, Pankhurst L, Hubbard A, Votintseva A, Stoesser N, Sheppard AE, Mathers A, Norris R, Navickaite I, Eaton C, Iqbal Z, Crook DW, Phan HTT. 2017. Resolving plasmid structures in Enterobacteriaceae using the MinION Nanopore sequencer: assessment of MinION and MinION/Illumina hybrid data assembly approaches. Microb Genomics 3:e000118. doi:10.1099/mgen.0.000118. PMC5610714 29026658

[8] Tatusova T, Dicuccio M, Badretdin A, Chetvernin V, Nawrocki EP, Zaslavsky L, Lomsadze A, Pruitt KD, Borodovsky M, Ostell J. 2016. NCBI Prokaryotic Genome Annotation Pipeline. Nucleic Acids Res 44:6614–6624. doi:10.1093/nar/gkw569. 27342282 PMC5001611

[9] Bortolaia V, Kaas RS, Ruppe E, Roberts MC, Schwarz S, Cattoir V, Philippon A, Allesoe RL, Rebelo AR, Florensa AF, Fagelhauer L, Chakraborty T, Neumann B, Werner G, Bender JK, Stingl K, Nguyen M, Coppens J, Xavier BB, Malhotra-Kumar S, Westh H, Pinholt M, Anjum MF, Duggett NA, Kempf I, Nykäsenoja S, Olkkola S, Wieczorek K, Amaro A, Clemente L, Mossong J, Losch S, Ragimbeau C, Lund O, Aarestrup FM. 2020. ResFinder 4.0 for predictions of phenotypes from genotypes. J Antimicrob Chemother 75:3491–3500. doi:10.1093/jac/dkaa345. 32780112 PMC7662176

[10] Zankari E, Allesøe R, Joensen KG, Cavaco LM, Lund O, Aarestrup FM. 2017. PointFinder: a novel Web tool for WGS-based detection of antimicrobial resistance associated with chromosomal point mutations in bacterial pathogens. J Antimicrob Chemother 72:2764–2768. doi:10.1093/jac/dkx217. 29091202 PMC5890747

